# Thermostable designed ankyrin repeat proteins (DARPins) as building blocks for innovative drugs

**DOI:** 10.1016/j.jbc.2021.101403

**Published:** 2021-11-15

**Authors:** Johannes Schilling, Christian Jost, Ioana Mariuca Ilie, Joachim Schnabl, Oralea Buechi, Rohan S. Eapen, Rafaela Truffer, Amedeo Caflisch, Patrik Forrer

**Affiliations:** 1 Athebio AG, Zürich-Schlieren, Switzerland; 2 Department of Biochemistry, University of Zürich, Zürich, Switzerland

**Keywords:** designed ankyrin repeat protein, DARPin, drug development, ensovibep, abicipar pegol, thermostability, N-terminal capping repeat, drug engineering, molecular dynamics, DARPins, designed ankyrin repeat proteins, HER2, human epidermal growth factor receptor 2, hGABP, human guanine-adenine-binding protein, HSA, human serum albumin, IR, internal repeat, VEGF, vascular endothelial growth factor

## Abstract

Designed ankyrin repeat proteins (DARPins) are antibody mimetics with high and mostly unexplored potential in drug development. By using in silico analysis and a rationally guided Ala scanning, we identified position 17 of the N-terminal capping repeat to play a key role in overall protein thermostability. The melting temperature of a DARPin domain with a single full-consensus internal repeat was increased by 8 °C to 10 °C when Asp17 was replaced by Leu, Val, Ile, Met, Ala, or Thr. We then transferred the Asp17Leu mutation to various backgrounds, including clinically validated DARPin domains, such as the vascular endothelial growth factor-binding domain of the DARPin abicipar pegol. In all cases, these proteins showed improvements in the thermostability on the order of 8 °C to 16 °C, suggesting the replacement of Asp17 could be generically applicable to this drug class. Molecular dynamics simulations showed that the Asp17Leu mutation reduces electrostatic repulsion and improves van-der-Waals packing, rendering the DARPin domain less flexible and more stable. Interestingly, this beneficial Asp17Leu mutation is present in the N-terminal caps of three of the five DARPin domains of ensovibep, a SARS-CoV-2 entry inhibitor currently in clinical development, indicating this mutation could be partly responsible for the very high melting temperature (>90 °C) of this promising anti-COVID-19 drug. Overall, such N-terminal capping repeats with increased thermostability seem to be beneficial for the development of innovative drugs based on DARPins.

Designed ankyrin repeat proteins (DARPins) are a class of antibody mimetics that have been conceived and developed about two decades ago at the University of Zurich (1, 2, 3). Their application as research tool and protein therapeutic was recently reviewed (4). Originally devised as an alternative to immunoglobulins (“antibodies”), the potential of DARPins in protein engineering, directed evolution of binders, and drug development became obvious immediately at inception. Importantly, this potential extends beyond areas of applications that have classically been “occupied” by recombinant immunoglobulins. The DARPin scaffold was shown to serve as an alternative (5), as a complementation, (6) and as an expansion of what is possible with binders derived from immunoglobulins (7, 8). Translation of academic research in DARPin technology toward pharmaceutical benefits has been predominantly steered by Molecular Partners, who provided the fundamental clinical validation of the scaffold (9). However, in light of the long generation cycles in drug development—especially in the case of biologics that typically require 10 years from concept to drug approval—the DARPin technology can still be regarded as young and emerging, and the full potential of DARPins as a class of biologics has yet to be realized. The recent development of ensovibep (10), a multi-specific anti-SARS-CoV-2 DARPin, which has entered clinical trials in November 2020 in less than 9 months after initial research and development activities had commenced, reinforces this high potential (11, 12).

DARPins are based on natural ankyrin repeat proteins (13) that have evolved to mediate various kinds of protein-protein interactions in all kingdoms of life (14). Their structure is simpler than that of immunoglobulins. Immunoglobulins naturally consist of four polypeptide chains and unite more than four chains in recombinant formats like T-cell bispecifics (15), whereas a single polypeptide chain is sufficient to form a multispecific DARPin (16). For example, ensovibep (10) combines five DARPin domains on a single polypeptide chain, in which two domains bind human serum albumin (HSA) and three domains associate with the SARS-CoV-2 spike protein (11). DARPins are built from solenoid protein domains, which possess a modular architecture that was derived by a consensus design approach (2, 17, 18): a stack of internal ankyrin repeats, each composed of 33 amino acids, flanked by N- and C-terminal capping repeats (N- and C-Caps) that function to seal the hydrophobic core of the protein domain (Fig. 1). Together, these structural units form an elongated ankyrin repeat domain. Amino acids present at defined positions at the surface of the internal repeats form a paratope, enabling the binding to target proteins with high affinity and specificity (3, 19, 20). These positions are randomized in DARPin libraries, which are used as starting point for *in vitro* selection, most prominently by means of ribosome display (21), to generate highly specific target-binding molecules. Originally, the N- and C-Caps of DARPins were taken from the human guanine-adenine-binding protein (hGABP_beta1) as they could be adapted to fit to the consensus-designed internal repeats (2). Such original N- and C-Caps are also present in the first DARPin that became clinically validated abicipar pegol. Despite the clinical validation, most of the amino acid sequence of these original caps was not optimized, indicating that there may be room for further improvements, in particular, for those that increase DARPin thermostability.

**Figure 1:**
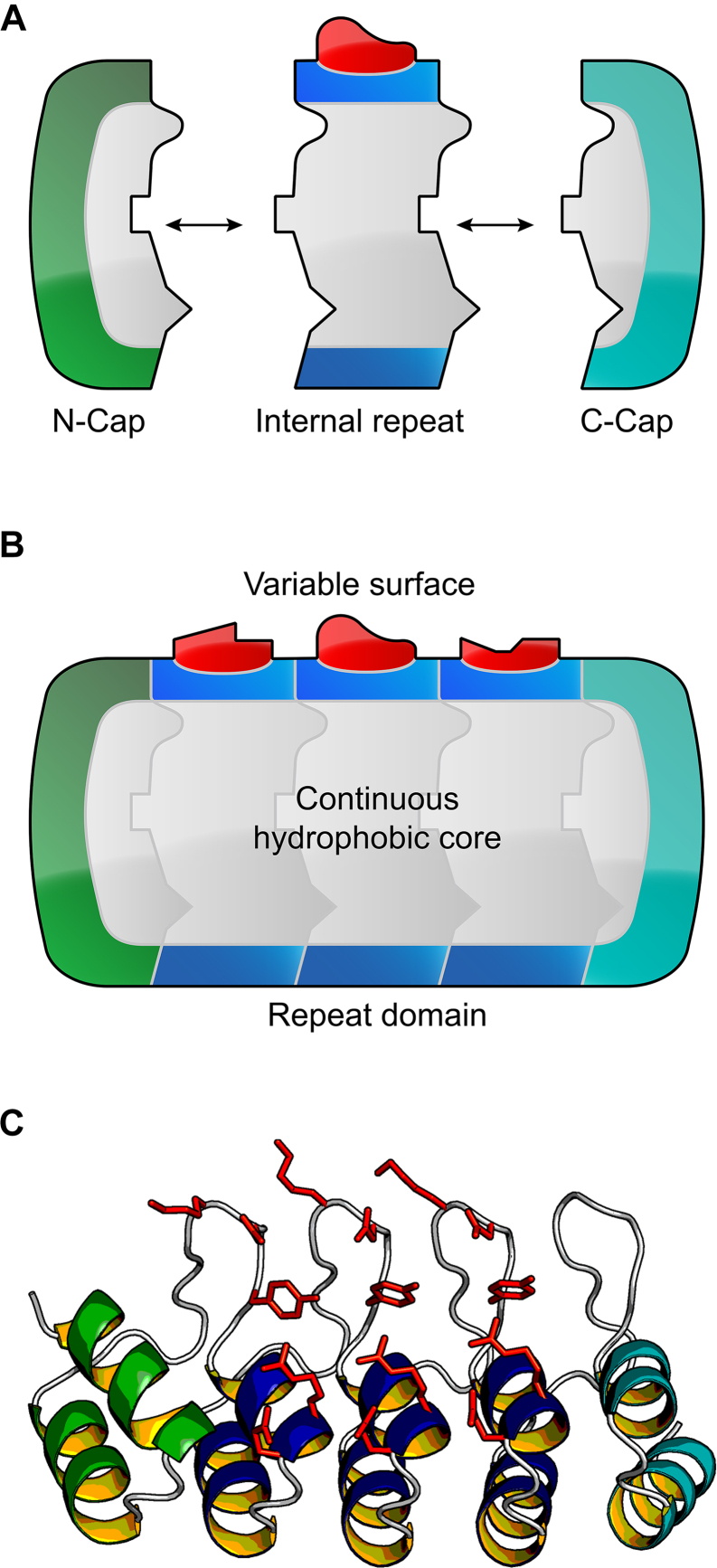
**Generic DARPin representation.***A*, schematic representation of a DARPin domain. The designed N- and C-terminal capping repeats and designed self-compatible repeat modules are the building blocks that stack *via* compatible interfaces. *B*, the repeat modules stack together forming a continuous hydrophobic core, which is sealed by an N-Cap (*green*) and a C-Cap (*cyan*). The repeat domains display variable molecular surfaces (randomized positions, depicted in *red*), which are potential target-binding sites (1, 2). *C*, structure of a DARPin (PDB ID: 2XEE (25)) in ribbon representation with the same color scheme as above: helices of the N-terminal capping repeat, the internal repeat modules, and the C-terminal capping repeat are colored *green*, *blue*, and *cyan*, respectively. The side chains of randomized positions are displayed as *red sticks*. This figure was created with open-source PyMOL (https://github.com/schrodinger/pymol-open-source). DARPin, designed ankyrin repeat protein.

In general, we identify three major motivations that fuel the quest for increased thermostability of biologics: (i) reduction of aggregation and thus reduction of immunogenicity risk, (ii) simplification of chemistry, manufacturing, and controls processes, thus bringing down the manufacturing costs and reduction of cold chain drug storage requirements, and (iii) increase of degrees of freedom for protein engineering to allow for mode-of-action design, for example, advanced multispecificity (11), receptor fine-tuning (22), and proximity-based activation (23). For the thermostability of DARPins, the importance of the capping repeats—particularly the C-Cap—was first shown by Interlandi *et al.* (24). Seven point mutations, five of which are located at the interface to the preceding internal repeat and two at the very C-terminus, were introduced to optimize the C-Cap and were shown to increase the *T*_m_ of a model DARPin, consisting of an N-Cap, a single full-consensus repeat and a C-Cap by about 17 °C; that is, from 60 °C (wt) to 77 °C (the respective C-Cap variant was referred to as the “mut5” C-Cap) (24). When this improved mut5 C-Cap was compared with the original C-Cap (2), a rigid-body movement of the C-Cap toward the internal repeat was observed, as evidenced by crystallographic data (25). This movement results in an increased buried surface area and a superior complementarity of the interface between the internal repeat and the C-Cap, which explains the improved thermostability.

Although the C-Cap of DARPins was thoroughly investigated, we are not aware of any scientific article describing a corresponding analysis or thermostability improvement of the original N-Cap (denoted as “N01” N-Cap in the following, (Fig. 2)), which is still predominantly used by the research community. Nevertheless, thermostability improvements of the N-Cap have been published in the patent application WO2012/069655 (WO′655) and are clinically validated in the aHSA domains of ensovibep (10). The corresponding N-Cap of WO′655 is denoted as “N02” in the following (Fig. 2). Comparing N02 with the original N-Cap, N01, resulted in a *T*_m_ increase of approximately 7 °C. It is likely that this improvement mainly arises from the Met24Leu-mutation present in N02, which removes the only methionine (besides the methionine encoded by the start codon) from the original DARPin sequence, and thereby also removes this hotspot for oxidation. The N-Cap analysis of WO′655 was limited to the RILMAN sequence motif around Met24 of N01.

**Figure 2:**
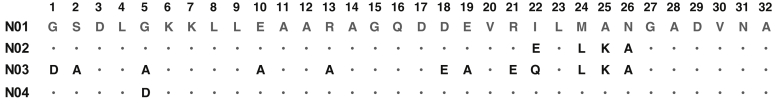
**Amino acid sequence alignment of different DARPin N-Caps.** Amino acids in N02, N03, and N04 that are identical to the one present in N01 are indicated as *dots*. The sequence numbering is shown as used in the text. N01, the original N-Cap, as described by Binz *et al*. (2). N02, an improved N-Cap, as described in WO′655 and present in the two anti-HSA DARPin domains of ensovibep (10). N03, an N-Cap where 12 out of the 32 amino acids are changed in comparison to N01. N04, the N-Cap of the DARPin domain of abicipar pegol (28). DARPin, designed ankyrin repeat protein; HSA, human serum albumin.

Here, we set out to improve the thermostability of DARPins through engineering the N-Cap. Through in silico analyses and high-temperature unfolding experiments at equilibrium, we identify N-Cap Asp17 as an Achilles heel of DARPin domains. Molecular dynamics (MD) simulations and MD trajectory analysis provide an explanation for the significantly increased *T*_m_ values observed upon Asp17 replacement in DARPin domains.

## Results

### Choice of a minimal DARPin as model system

We chose a three repeat DARPin domain (denoted as N1C in the following) consisting of one full-consensus internal repeat (IR) flanked by an N- and C-Cap as a model DARPin to screen for improved thermostability (see Table 1 for an overview of the different DARPin domains used, as well as Table S1 for their respective sequences). The choice of this DARPin model has a 3-fold motivation. First, it has the minimal DARPin architecture consisting of only three repeats with two repeat interfaces, one between the N-Cap and the IR and one between the IR and the C-Cap. Second, using a consensus IR that represents an “average structure” of all the natural ankyrin repeats should eliminate interferences originating from amino acids at randomized positions or possible framework mutations, that may only be present in particular sequences (18). Third, because DARPins get more stable with increasing number of IRs, we choose to have only one internal repeat and thus a low starting thermostability (26) such that stability improvements are readily observable.

**Table 1:** Description of DARPin domains used in the present study with varying N- and C-Caps, as detailed in the text

Domain name	Description
N1C	DARPin domain with one full consensus IR corresponding to NI1C of Interlandi *et al.* (24) & Wetzel *et al.* (26).
aHER2	DARPin domain based on an anti-HER2 DARPin domain corresponding to H10-2-G3 (“G3”; Zahnd *et al.* (20)).
aVEGF	DARPin domain based on an anti-VEGF-A DARPin domain of the protein moiety of abicipar pegol (28).
aHSA	DARPin domain based on the anti-HSA DARPin domain of ensovibep (10). It possesses an improved C-Cap similar to the mut5 C-Cap described by Interlandi *et al.* (24).

For a comprehensive list of amino acid sequences of all domain variants used, see Table S1.

### Importance of the N-Cap position 17 for the overall thermostability of DARPins

**Figure 3:**
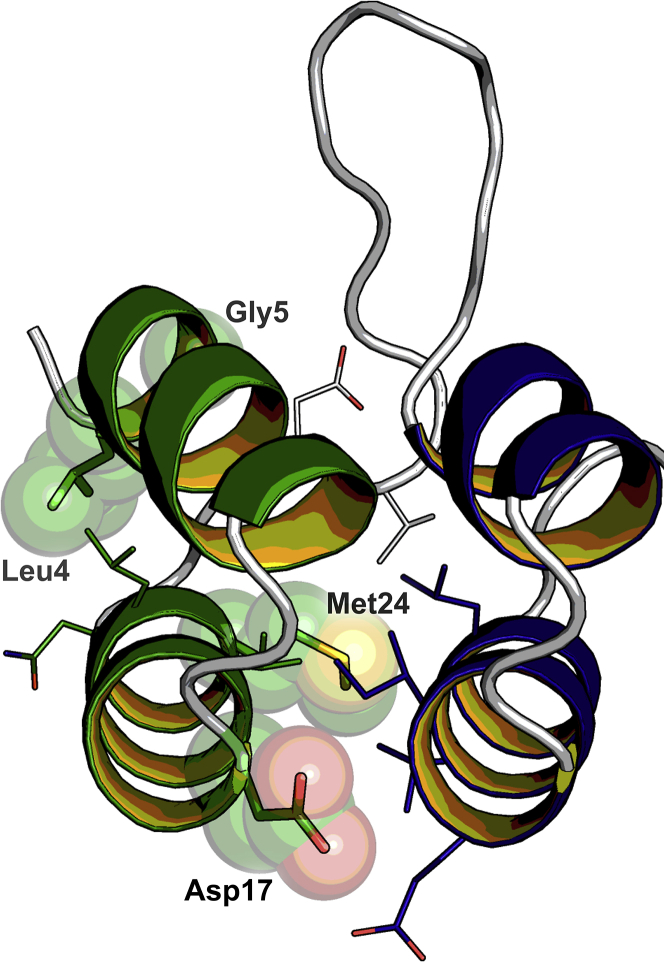
**Ribbon diagram of an N01 N-Cap (*green*) and first IR (*blue*) of a conventional DARPin (PDB ID:****2XEE** (25)**).** The side chains of N-Cap residues Leu4, Gly5, Asp17, and Met24 are displayed as *spheres* and *sticks*, and the surrounding side chains are displayed as *lines*. This figure was created with open-source PyMOL (https://github.com/schrodinger/pymol-open-source). DARPin, designed ankyrin repeat protein; IR, internal repeat.

**Figure 4:**
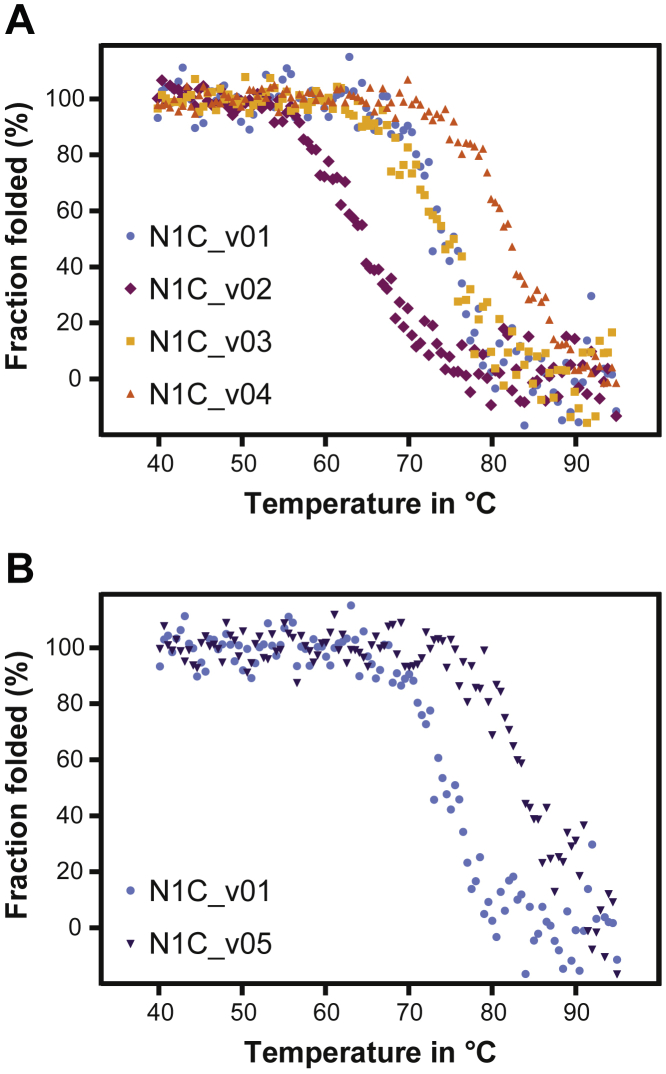
**Thermal unfolding of DARPin domains followed by CD spectroscopy between 40 °C and 95 °C; all variants were measured at a concentration of 10 μM in PBS.***A*, N1C_v01 to N1C_v04: The measured T _m_ values were 74.5 °C for the control N1C (N1C_v01), 64.7 °C for the N-Cap Leu4Ala mutant (N1C_v02), 74.3 °C for the N-Cap Gly5Ala mutant (N1C_v03), and 82.4 °C for the N-Cap Asp17Ala mutant (N1C_v04). *B*, the Asp17Leu mutant (N1C_v05) has a T _m_ of 84.6 °C and is strongly stabilized compared with N1C_v01 containing Asp17. DARPin, designed ankyrin repeat protein.

By visual analysis of the DARPin structure, we identified four residues within the N-Cap to be of potential importance for the repeat stacking and thereby also for the overall domain stability. These residues are either at the edge (Leu4, Gly5, and Asp17) of the N-Cap or buried (Met24) at the interface between N-Cap and the adjacent IR (Fig. 3). As WO′655 already demonstrated that a Met24Leu mutation strongly improves the thermostability of DARPins, we focused our analysis on Leu4, Gly5, and Asp17 on an N1C background comprising the N02 N-Cap to find out if it is possible to further improve the most stable N-Cap known to date. Alanine scanning of residues four and five showed no improvement in thermostability, with Leu4Ala lowering the *T*_m_ value from 74.5 °C to 64.7 °C and Gly5Ala leaving the melting temperature unaltered at 74.3 °C. However, the Asp17Ala mutation showed a strong improvement of the *T*_m_ value from 74.5 °C to 82.4 °C (Fig. 4*A*). Consequently, we screened alternative amino acids at the N-Cap position 17 of N1C (Table 2 and Fig. 4*B*). All amino acid substitutions tested (excluding *e.g.*, Cys, Trp, and Gly, that would not make sense from the point of protein engineering) resulted in a *T*_m_ increase, with the highest *T*_m_ increases being measured for the Asp17Val, Asp17Ile, and Asp17Leu variants (*i.e.*, from 74.5 °C to 85.1 °C, 84.8 °C, and 84.6 °C, respectively). Overall, changing Asp17 in N1C to Val, Leu, Ile, Met, Ala, or Thr led to an increase of the respective *T*_m_ values between 8 °C to 10 °C. These results show that Asp is an exceptionally unfavorable amino acid at position 17, and that there are many alternative residues resulting in a strong thermostability gain. Of these alternative residues, Asp17Leu provided one of the largest improvements, which we investigated further.

**Table 2:** *T*_*m*_ values of N1C variants having various amino acids at position 17

Name	Position 17	*T*_m_ (°C)
N1C_v01	D	74.5
N1C_v04	A	82.4
N1C_v05	L	84.6
N1C_v06	V	85.1
N1C_v07	M	83.8
N1C_v08	I	84.8
N1C_v09	T	82.3
N1C_v10	S	79.3
N1C_v11	N	75.2
N1C_v12	Q	77.4
N1C_v13	K	77.9
N1C_v14	R	78.3
N1C_v15	E	79.2

### The increased thermostability of the Asp17Leu N-Cap mutation is independent of the N- and C-Cap background

To test if the improvements derived from mutating the N-Cap Asp17 are generic and independent of the N02 background, we transferred the Asp17Leu mutation onto the original N01 N-Cap and the N03 N-Cap that differs in nine amino acids from N02. The Asp17Leu mutation improved the thermostability of N1C also in the N01 and N03 backgrounds by more than 13 °C (Table 3). Further, we were interested in whether our observed stability improvement based on the N-Cap and the stability improvement based on the mut5 C-Cap (24) would be additive. We first found that replacing the wt C-Cap in N1C with a mut5 C-Cap results in a *T*_m_ increase of about 13 °C or 9 °C in an N01 or N02 background, respectively (Table 4), thus confirming the benefits of the mut5 C-Cap (24). In addition, the combination of the N02 N-Cap with the mut5 C-Cap in N1C_v22 proved that the individual improvements of each cap are additive and raised the *T*_m_ value to 84.2 °C in PBS, that is, by about 22 °C. With the additional substitution of Asp17Leu in N1C_v23 (N02, mut5 background), we did not observe any unfolding transition up to 95 °C when we measured the thermal unfolding of this molecule in PBS. Therefore, we repeated the measurements for N1C_v22 and N1C_v23 in a buffer containing 2 M GdmCl and obtained corresponding *T*_m_ values of 67.3 °C and 79.3 °C, respectively (Table 4). Thus, the already very thermostable N1C_v22, comprising N02 and the mut5 C-Cap, could be further stabilized by adding the Asp17Leu mutation to its N-Cap resulting in a *T*_m_ gain of about 12 °C in 2 M GdmCl. Overall, the Asp17Leu mutation adds about 9 °C to 14 °C to the *T*_m_ value of N1C independent of its concrete N- and/or C-Cap, indicating that this is a general improvement for DARPin domains.

**Table 3:** *T*_*m*_ values of N1C variants having either Asp or Leu at position 17 of the N-Cap in N01, N02, or N03 backgrounds

Name	N-Cap	C-Cap	*T*_m_ (°C)
N1C_v01	N02	wt	74.5
N1C_v05	N02_D17L	wt	84.6
N1C_v16	N01	wt	62.1
N1C_v17	N01_D17L	wt	75.2
N1C_v19	N03	wt	68.6
N1C_v20	N03_D17L	wt	82.8

**Table 4:** *T*_*m*_ values of N1C variants having either Asp or Leu at position 17 of the N-Cap in wt and mut5 C-Cap backgrounds

Name	N-Cap	C-Cap	*T*_m_ (°C)	*T*_m_^a^ (°C)
N1C_v16	N01	wt	62.1	ND
N1C_v25	N01	mut5	74.6	ND
N1C_v01	N02	wt	74.5	ND
N1C_v22	N02	mut5	84.2	67.3
N1C_v23	N02_D17L	mut5	ND	79.3

Abbreviation: ND, not determined.

a Indicates *T*_*m*_ measurements in 2 M GdmCl.

### MD simulations suggest reduced flexibility of N1C through the Asp17Leu N-Cap mutation

**Figure 5:**
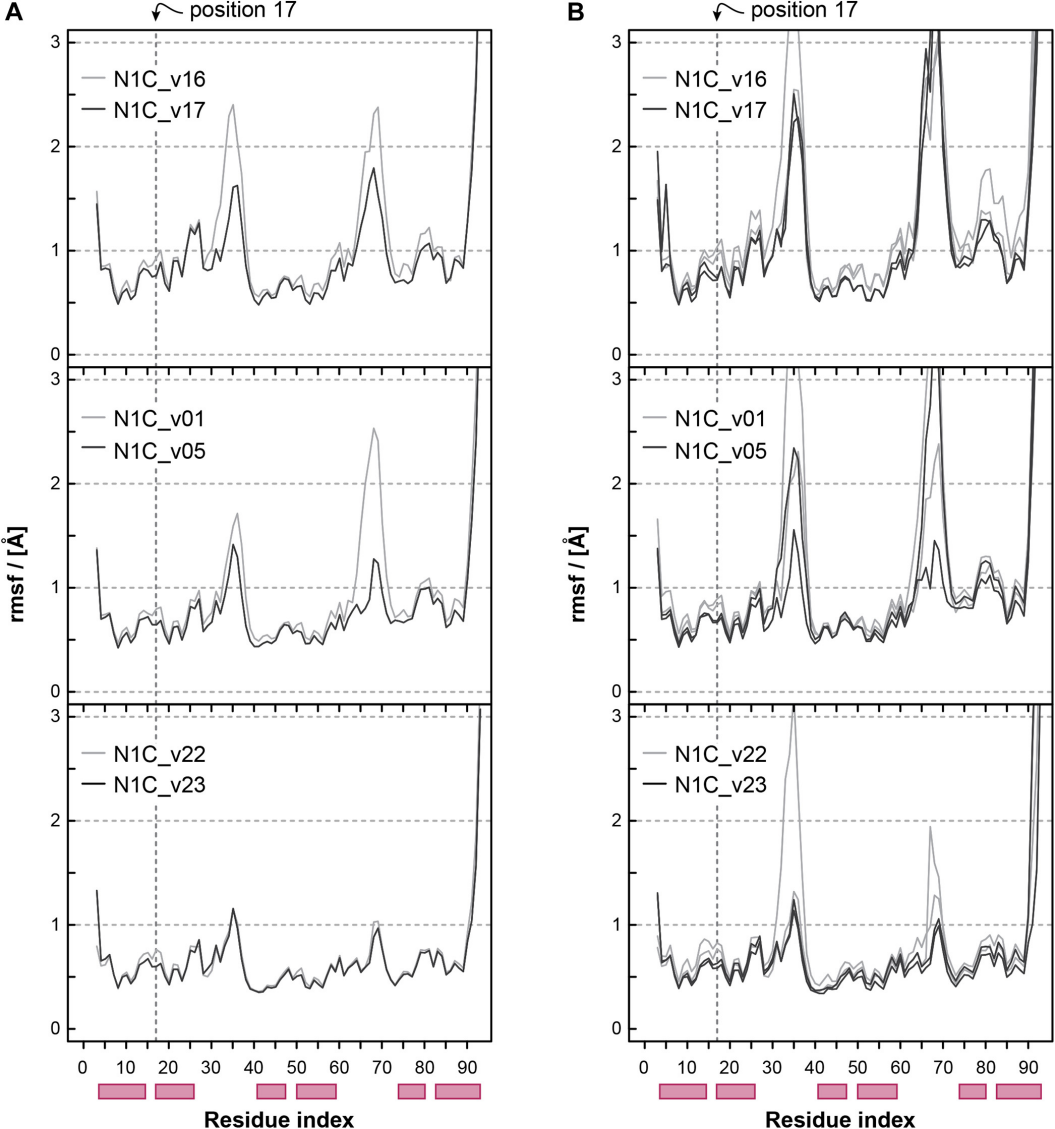
**RMSF profiles of the proteins at 400 K.** Compared are the Asp17 (*light gray*) with the Leu17 (*black*) constructs in the N01-background (*top*), the N02-background (*middle*), and the N02-background combined with the mut5 C-Cap (*bottom*). Two different timescales are shown comprising (*a*) fluctuations over 5 ns and (*b*) 150 ns. The RMSF profiles on the 5 ns timescale were calculated as the average over 30 independent 5-ns profiles. The four independent profiles for the 150 ns timescales show the fluctuations over the first and the last 150 ns of the production simulations that is, the last 300 ns of the 600 ns run. The fluctuations were calculated about the average structure determined from the corresponding 5 ns or 150 ns intervals. The stability of the folded structure of the Leu17-constructs is higher than the Asp17-constructs as the former show lower Cα RMSF. The helical regions of the secondary structure are indicated by *red boxes* below the residue index.

We performed MD simulations to investigate the structural implications for the N1C variants having either Asp or Leu at position 17 of their N-Caps. Starting from the X-ray diffraction structure of ankyrin repeat proteins of E3_5, NI1C-mut4, and NI3C-mut5 (PDB ID: 1MJ0 (27), 2XEN (25), and 2XEE (25), respectively), we prepared six different homology models, each time comparing Asp17 with Leu17 constructs in the N01-background (N1C_v16 and N1C_v17, respectively), the N02-background (N1C_v01 and N1C_v05, respectively), and the N02-background combined with the mut5 C-Cap (N1C_v22 and N1C_v23, respectively) (see Table S1). Of note, the numbering in PDBs may be different for different DARPins, which is because of the fact that some PDB structures' counting might for example, include N-terminal tags like the MRGSH6-tag used for purification. In the above mentioned PDB ID entries, amino acid numbers corresponding to the N-Cap position 17 are #27 for 1MJ0 and #15 for 2XEN and 2XEE. Starting from the homology models, three independent simulations were carried out for each system, two at 350 K and one at 400 K, for a total sampling of 1.8 μs. Three conclusions can be drawn from the MD simulations (Fig. 5 and Table 5). First, the substitution of Asp at position 17 with Leu leads in all instances to improved interaction energies with the surrounding (Table 5). Second, the analysis focused on the protein flexibility at high temperatures (400 K) and on two different timescales (5 ns and 150 ns) revealed that the systems containing Leu at position 17 systematically show lower fluctuations than their Asp17 counterparts (Fig. 5). The effects of the Asp17Leu mutation are more pronounced on the 150-ns timescale (Fig. 5*B*) than on the 5-ns timescale (Fig. 5*A*). The profiles along the full protein sequences show the least fluctuations in the helical segments and the highest flexibility at the loops. The higher rigidity of the helical segments of the Leu17 mutants (lower fluctuations) is relevant for enthalpic stabilization. The differences in the fluctuations of the loops are less relevant as their flexibility contributes to entropic stabilization. The reduced flexibility of the Leu17 mutants as compared with Asp17 is in line with the increased thermostability of Asp17Leu DARPins observed in CD. Third, on the longer timescale, the Asp17Leu mutation reduces fluctuations in the N01-background (N1C_v16 *versus* N1C_v17) across the entire N-Cap and in one of the most flexible parts of a DARPin domain spanning from the end of the N-Cap (GADVNA motif, residues 27–32 (Fig. 2)) to the β-turn at the beginning of the internal repeat. In the N02-background (N1C_v01 *versus* N1C_v05) and the mut5 C-Cap (N1C_v22 *versus* N1C_v23), reduced fluctuations through the Asp17Leu mutation are more pronounced in the direct vicinity of position 17. The localized stability may be related to the stabilizing Met24Leu mutation of N02. Furthermore, the least fluctuations along the protein sequence are observed in the background comprising N02 and the mut5 C-Cap (N1C_v22 and N1C_v23), which may be explained by the stabilizing effects introduced by the improved mut5 C-Cap. These findings further support our hypothesis that position 17 is an Achilles heel with respect to the N-Cap and thus the overall DARPin domain thermostability.

**Table 5:** Interaction energies between residue 17 and the rest of the protein

Name	Coulombic (kcal/mol)		van-der-Waals (kcal/mol)		Total energy (kcal/mol)		*T*_*m*_ (°C)
	Run 1	Run 2	Run 1	Run 2	Run 1	Run 2	
N1C_v16	−105.8	−104.8	−8.4	−8.2	−114.2	−113.0	62.1
N1C_v17	−109.1	−109.5	−10.5	−10.4	−119.6	−119.9	75.2
N1C_v01	−107.5	−106.2	−8.2	−8.3	−115.7	−114.5	74.5
N1C_v05	−109.3	−109.2	−10.2	−10.3	−119.5	−119.5	84.6
N1C_v22	−106.7	−105.5	−8.4	−8.2	−115.1	−113.8	67.3^a^
N1C_v23	−109.4	−109.5	−10.3	−10.3	−119.7	−119.8	79.3^a^

The analysis was performed on the last 300 ns of each run at T = 350 K.

a Indicates *T*_*m*_ measurements in 2 M GdmCl.

### The Asp17Leu N-Cap mutation improves the stability of clinically validated DARPins

To test whether the observed thermostability gain derived from the N-Cap Asp17Leu is independent on the composition of randomized positions in the DARPin paratope (as mainly present in the IRs) and thus transferable to any (nonconsensus) DARPin, we tested this mutation on binders selected against human epidermal growth factor receptor 2 (HER2) (20), vascular endothelial growth factor A (VEGF-A) (28), and HSA (29) (Table 6). The selected DARPin domains are denoted aHER2, aVEGF (which carries a Gly5Asp framework mutation in its N-Cap; denoted as N04 in (Fig. 2)), and aHSA, respectively (Table 1). Both aVEGF and aHSA are clinically validated DARPin domains, as they are present in abicipar pegol and ensovibep, respectively. In all the three transfers, the Asp17Leu mutation increased the thermostability of the DARPin domains and added up to about 15 °C in 2 M GdmCl *T*_m_ measurements (Table 6). Overall, the described significant gain in thermostability of the Asp17Leu mutation proved to be generic and is transferable to different N- and C-Cap backgrounds and different library members selected for high affinity, including clinically validated DARPin domains.

**Table 6:** *T*_*m*_ values of DARPin variants specifically binding to VEGF, HER2, and HSA

Name	N-Cap	*T*_*m*_ (°C) (in 2 M GdmCl)
aVEGF_v01	N04	Unfolded at RT
aVEGF_v02	N04_D17L	45.1
aHER2_v01	N01	39.6
aHER2_v02	N01_D17L	55.7
aHSA_v01	N02	76.0
aHSA_v01	N02_D17L	84.1

Abbreviation: RT, room temperature.

## Discussion

The results presented in this paper identify position 17 of the DARPin N-Cap as a key position influencing thermostability of DARPins. The importance of the capping repeats as a prerequisite for the high stability and robustness of the DARPin-fold has been known since the first description of this antibody mimetic (26, 27). Although the C-Cap has been thoroughly analyzed for residues providing room for improvement previously (24), an analogous dissection of the N-Cap in the literature, with exception of WO′655, has been missing.

### The N-Cap contributes significantly to the thermostability of DARPins

The contribution of the capping repeats to the overall stability of the DARPin domain is central (26). The architecture of solenoid repeat protein domains does not rely on long-range interactions (distant in sequence), which is a fundamental difference to globular proteins. In repeat proteins, stabilizing and structure-determining interactions are formed within a repeat and between neighboring repeats. In contrast to IRs, the capping repeats have only one stabilizing neighboring repeat (Fig. 1). Both natural (30) and designed ankyrin repeat proteins (26) have been shown to unfold in a sequential manner starting with the capping repeats. An unfolded cap leads to an internal repeat losing a stabilizing neighbor resulting in destabilization of the whole repeat domain. Furthermore, the original N- and C-Caps of DARPins correspond mainly to the natural caps of hGABP_beta1 (2); and thus there may be room for improvements. Indeed, improving the original capping repeats can increase the overall stability of a DARPin domain, as it was shown for the N-Cap (WO′655) and the C-Cap (24). The improved N02 N-Cap of WO′655 comprises a Met24Leu mutation when compared with N01 (Table 2), thereby improving the thermostability of DARPin domains. Because Met possesses a larger side chain than Leu, it might not optimally fit into the confined space at the interface between the N-Cap and the adjacent internal repeat. This mutation has the additional advantage that it eliminates an oxidation hotspot from the DARPin domain; unwanted oxidation of Met and its negative impact on the bioactivity of biologics is well documented (31).

We now performed a detailed analysis of the N-Cap, searching for additional mutations that may improve the overall DARPin domain stability. Following *in silico* analyses, we used equilibrium thermal denaturation experiments of DARPins harboring different N-Cap mutations. Intriguingly, our studies show that position 17 of the N-Cap is an important Achilles heel of a DARPin domain and that the original Asp at position 17 of the N-Cap is detrimental to overall DARPin thermostability. We showed that the negative effect on thermostability of Asp17 can be rescued by mutating it to Leu, Val, Ile, Ala, Met, or Thr, leading to a profound improvement in *T*_m_ values by about 8 °C to 16 °C depending on the individual construct tested. Importantly, the *T*_m_ value of an N1C variant comprising the N02 N-Cap (instead of N01) was also increased by about 9 °C when introducing the Asp17Leu mutation. Thus, the beneficial effect of this mutation synergizes with the *T*_m_ improvement of about 7 °C previously reported for N02 (WO′655). Although Interlandi *et al*. (24) reported that the C-Cap is a limiting factor for the thermostability of the original DARPin domain, our results demonstrate that this is also the case for the N-Cap.

### Asp17 replacements seem to improve the N-Cap/IR interface

**Figure 6:**
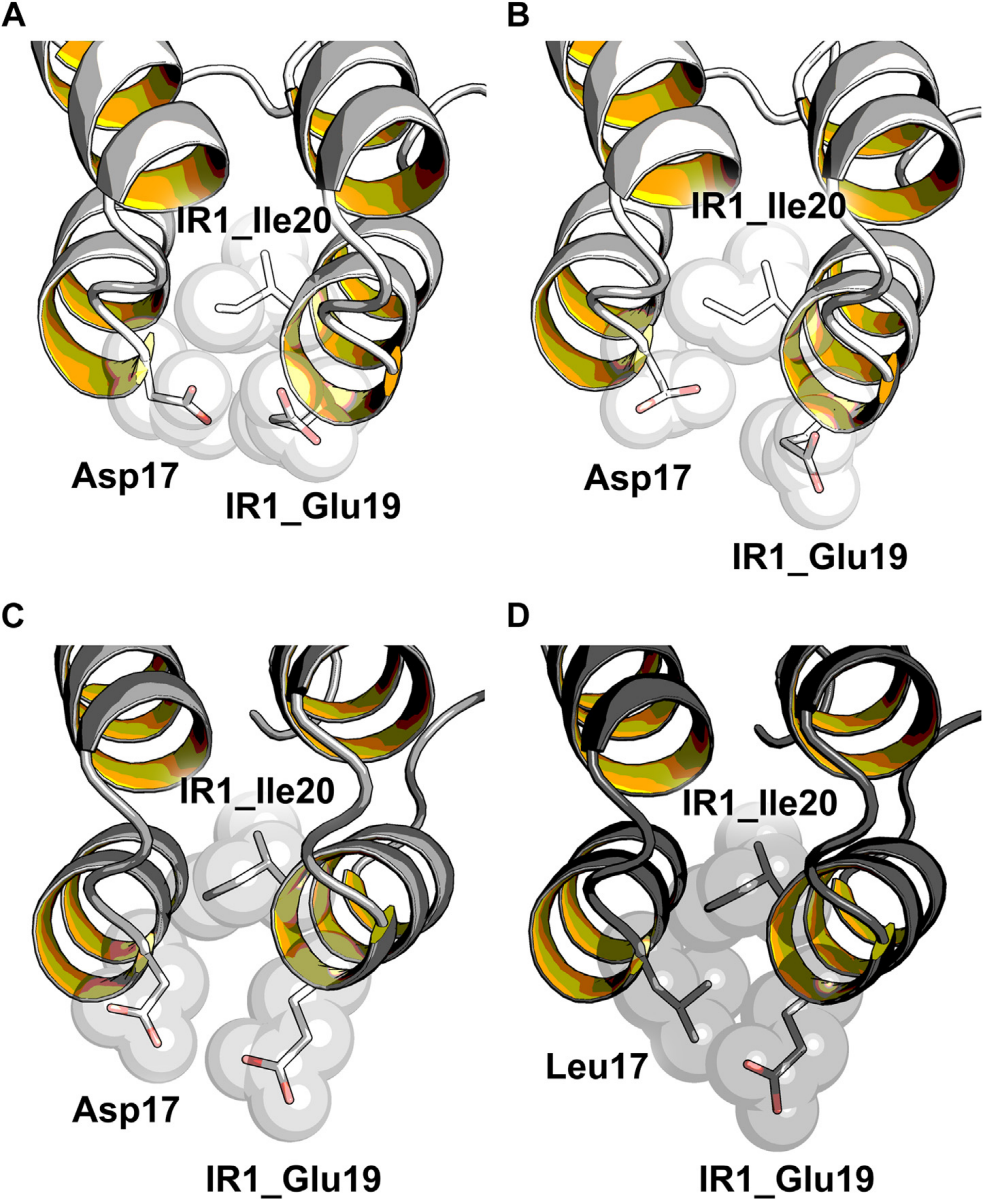
**Side chain orientationsand interactionsof Asp17 and Leu17 with surrounding residues.***A*, inward and (*B*) outward facing Asp17 present in 2V4H (46). The average structures of the systems equilibrated at 300 K with (*C*) outward facing Asp17 and (*D*) inward facing Leu17. The N-Cap position 17 and internal repeat positions IR1_Glu19 and IR1_Ile20 are displayed as *spheres* and *sticks*. This figure was created with open-source PyMOL (https://github.com/schrodinger/pymol-open-source).

The N-Cap position 17 is located at the beginning of helix two at the edge of the interface between the N-Cap and the adjacent IR (IR1) (Fig. 6*A*). Throughout nature, the negatively charged amino acids are found at the N-terminal turn of helices as they support helix formation during protein folding (32), which rationalizes the placement of Asp at position 17. The known DARPin crystal structures show that the side chain of Asp17 can either face inward and be buried in the interface between N-Cap and IR1, or face outward into the surrounding solvent. If Asp17 is facing inward, it is involved in van-der-Waals contacts with IR1 (*i.e.*, to the side chains and backbones of IR1 Glu19 and IR1 Ile20), but is burdened with a desolvation penalty, which negatively impacts thermodynamic stability (Fig. 6*A*). If facing outward, Asp17 shields the hydrophobic interface from the solvent, but experiences repulsive forces to the equally negatively charged IR1 Glu19 (Fig. 6*B*). The different rotamers in different structures are consistent with the lack of favorable interactions of Asp17 with the rest of the protein (Table 5).

The analyses of equilibrated, energy-minimized average MD structures at 300 K and associated interactions between the N-Cap residue 17 and its surrounding at 350 K show that although in our starting model, Asp17 faced inward, during the course of the simulation, Asp17 (Fig. 6*C* and Table 5) is facing outward, as described above avoiding the desolvation penalty, but experiencing electrostatic repulsive forces to the IR1 Glu19. Importantly, the MD simulations show that Leu at position 17 faces inward and that the Asp17Leu mutation improves the Coulombic and the van-der-Waals interactions by about 3 kcal/mol and 2 kcal/mol, respectively (Fig. 6*D* and Table 5). The improved interactions of Leu17 are consistent with the strong increase in thermostability observed by CD measurements.

### The Asp17Leu N-Cap mutation is universally applicable to DARPins

Importantly, the observed stability improvement of about 8 °C to 16 °C (depending on the actual context) that was caused by the N-Cap Asp17Leu mutation proved to be generically applicable to various sequence backgrounds. First, as observed by CD measurements and supported through MD simulations, stabilization could be consistently achieved on a diverse set of N-Cap backgrounds grafted on the model DARPin N1C. The tested N-Cap backgrounds include the original N01, N02 of the two HSA-binding domains of ensovibep, N03 where 12 out of the 32 amino acids are changed in comparison to N01 and N04, present in the VEGF binding domain of abicipar pegol (Fig. 2). Second, the Asp17Leu mutation was also beneficial in the context of the improved mut5 C-Cap. Third, Asp17Leu gave a constant improvement when transferred to three different DARPin binders directed against HER2 (20), VEGF (28), or HSA (29), indicating that the observed profound improvement is independent of the randomized positions forming the DARPin paratope. This general transferability of the Asp17Leu mutation may be because of the fact that all DARPin domains are based on a quasi-identical framework embedding the randomized positions (27). The underlying reason for this similarity of the framework is the consensus design of DARPins, resulting in the stacking of self-compatible IRs (1, 2, 18). However, even the investigated DARPin domains aVEGF and aHER2 that possess framework mutations, which are key for their low picomolar binding affinities, still profit from the Asp17Leu mutation. This indicates that this single amino acid change may also be beneficial for DARPin domains comprising framework mutations; in particular, when these mutations have a negative effect on the domain stability.

The analyses of known X-ray structures of DARPins in complex with their respective targets have shown that N- and C-Caps contribute to the DARPin paratope in approximately 35% of the cases (33). Position 17 is located outside of these known paratope regions, and our MD based structural analyses indicate that the overall alteration of the domain structure through the Asp17Leu mutation is marginal. Thus, we do not expect that the N-Cap Asp17Leu change has a significant impact on the target binding of corresponding DARPin domains. Incorporation of the Asp17Leu mutation into existing DARPin binders could increase their thermostability without significantly impacting their target-binding properties.

### Quantitative dimension and additive nature of the Asp17Leu improvement

The benefit obtained by this novel N-Cap (*i.e.*, a *T*_m_ increase of 8 °C to 16 °C for a DARPin domain) is similar in dimension to that described for the C-Cap by Interlandi *et al*. (24). Most importantly, the N-Cap and C-Cap stability gains function in an additive manner, thereby yielding a *T*_m_ increase of approximately 20 °C when combined. Similarly, the stability improvement of the novel N-Cap mutation Asp17Leu is additive to the stability gain obtained through the known N-Cap mutation Met24Leu (WO′655). When compared with the original DARPins (1, 2), in sum, the two improvements at the N-Cap (each increasing the *T*_m_ value by approximately 10 °C) and the improvement at the C-Cap can thereby yield a total *T*_m_ increase of approximately 30 °C for a DARPin domain. Thus, our discovered Asp17Leu improvement adds substantially to other capping repeat improvements described previously. We see the modular, solenoid structure of DARPins as the origin for making the observed modular addition of thermostability improvements possible. DARPins are assembled through the stacking of repeat units that are structurally “self-organized”, yet connected. They are stabilized by interactions with their folded immediate neighboring repeats, and thus each repeat individually contributes to the overall domain stability (26).

### Ease of engineering of DARPins and impact on their versatility

DARPins unify a plethora of key characteristics beneficial for drug development from discovery to preclinical and clinical development and manufacturing. For example, their low molecular weight, high solubility, high expression level, picomolar affinities, multispecific potential, and especially the high stability offer drug developers access to huge versatility (9). With their simple modular structure, multispecific DARPins (16) can be easily constructed with a single polypeptide chain and different molecular formats, as the binding domains are freely combinable. Their high solubility and high expression level awards them with unparalleled high-throughput capabilities, and their excellent biophysical properties are the origin for low attrition rates. Their high thermostability, in particular, positively influences low aggregation and associated immunogenicity risks, allows for low-cost manufacturing and makes them very amenable to protein engineering to generate molecules with novel modes of action, such as receptor fine-tuning (22). With these characteristics, DARPins complement existing therapeutic antibodies and will also expand the scope of innovative drugs *via* the multitude of advanced formats and applications that can be conceived and realized with this scaffold. All mentioned characteristics build on the stability of this versatile scaffold. Thus, an increased thermostability as presented in this study will have a positive impact on many levels.

### Significance for clinical DARPins

A recent example of drug development at unparalleled speed is the development of the multi-specific, highly efficacious anti-SARS-CoV-2 DARPin ensovibep that went from first selection of binders to entry into the clinic in less than 9 months (12). Interestingly, ensovibep contains the Asp17Leu mutation described herein in three of its five DARPin domains (10). This not only gives clinical validation to the Asp17Leu mutation, but also underscores the importance and reach of the findings outlined here. The high thermostability (*T*_m_) of 90 °C and absence of any tendency for aggregation (up to 85 °C) reported for ensovibep (11) may thus be partly explained by the presence of the Asp17Leu mutation. These beneficial properties are especially remarkable as this COVID-19 antiviral drug is composed of five distinct DARPin domains with four different specificities on a single polypeptide chain. In our view, an immunoglobulin-based drug with similar properties would require extensive engineering to be generated, if feasible at all. Together, it does not surprise that Molecular Partners is speculating that ensovibep has the potential to bypass cold storage, and that it provides a superior alternative to monoclonal antibody cocktails for global supply (12). Besides the three DARPin domains binding to three unique epitopes of the spike ectodomain of SARS-CoV-2, that all carry Asp17Leu in their N-Caps, ensovibep is in addition composed of two anti-HSA DARPins for serum half-life extension. These two domains are identical to aHSA of our study. As suggested by our findings, the overall stability of ensovibep might be even further improved by replacement of Asp17 with Leu, Val, Ile, Ala, Met, or Thr in the N-Cap of the two anti-HSA DARPins.

Furthermore, aVEGF of our study corresponds to the ankyrin repeat domain of abicipar pegol that is a first generation DARPin drug to treat patients with neovascular age-related macular degeneration. Two randomized phase 3 clinical trials (CEDAR and SEQUOIA) demonstrated that quarterly applied abicipar pegol is noninferior to monthly applied ranibizumab, but showed a higher level of intraocular inflammation (typically mild or moderate in severity) than ranibizumab (34). The authors of these phase 3 studies mentioned that the used abicipar pegol was further purified to reduce host cell proteins with the goal to minimize the incidence of intraocular inflammation. As indicated by our findings, the thermostability of the ankyrin repeat domain of abicipar pegol could be increased by about 30 °C when combining our results with the previously described cap improvements. A correspondingly improved abicipar pegol would be amenable to more stringent purification processes that may result in much lower contaminations with host cell proteins.

## Conclusions

We have shown a *T*_m_ increase of various DARPin domains (up to 16 °C) by replacing Asp17 in the N-Cap with Leu, Val, Ile, Ala, Met, or Thr. Our results further provide evidence for the importance of the capping repeats for the robustness and reliability of this solenoid scaffold. Combining our N-Cap improvement with the cap enhancements described by Interlandi *et al*. (24) and WO′655 increases the melting temperature of original DARPin domains by about 30 °C. Even though the initial DARPin design (1, 2) is successfully used by the research community and is clinically validated (abicipar pegol), this significant improvement of the *T*_m_ indicates that the capping repeats of the original DARPins, which were based on hGABP_beta1, have high liabilities that can be eliminated by a few mutations in the capping repeats. Such thermodynamically stabilized antibody mimetics could pave the way for the future development of innovative drugs. First, increased thermostability of biologics is known to correlate with a reduced aggregation propensity (35). Thus, we anticipate an even lower immunogenicity risk of DARPins comprising the N-Caps described herein. Second, the improved thermostability will make DARPins even more amenable to protein engineering. We believe that this is of high importance when the biological activity of a drug needs to be optimized, for example, to fine-tune the activation of receptors (22). Third, the increased thermostability will translate into more efficient preclinical development and chemistry, manufacturing, and controls processes, low-cost manufacturing, and may even help to bypass the cold storage of biologics. The recent development of the DARPin ensovibep (comprising the N-Cap Asp17Leu mutation) in less than 9 months from idea conception to entry into the clinics underlines this huge potential (11, 12). Along the same lines, we speculate that a high thermostability may facilitate the development of aerosol or dry powder inhaler formulations of DARPin drugs; something that may also be of interest for the pulmonary delivery of ensovibep for the treatment of COVID-19 patients. In conclusion, future drug development asks for more robust and very versatile biologics, and we believe that DARPins would be the ideal scaffold for this.

## Experimental procedures

### Cloning

The DNA sequences encoding each ankyrin repeat domain were chemically synthesized and cloned into the pQIq expression vector (QIAGEN) by Gibson Assembly (36).

### Protein expression and purification

The ankyrin repeat domains were expressed in *Escherichia* *coli* XL1-blue cells and purified using their N-terminal MRGSH_6_-tag (encoded by the pQIq expression vector) by standard protocols. Briefly, 25 ml of stationary overnight cultures (LB, 1% glucose and 100 mg/l of ampicillin; 37 °C) were used to inoculate 1 L cultures (same medium). At an absorbance of about 1.0 at 600 nm, the cultures were induced with 0.5 mM IPTG and incubated at 37 °C for 4 h. The cultures were centrifuged, and the resulting pellets were resuspended in 40 ml of TBS500 (50 mM Tris–HCl and 500 mM NaCl, pH 8) and sonicated. The lysate was recentrifuged, and glycerol (10% (v/v) final concentration) and imidazole (20 mM final concentration) were added to the resulting supernatant. The ankyrin repeat domains were purified over a Ni-nitrilotriacetic acid column (2.5 ml column volume) according to the manufacturer's instructions (QIAGEN). Up to 200 mg of highly soluble ankyrin repeat domains were purified from 1 L of *E.* *coli* culture with a purity >95% as estimated from SDS-PAGE.

### CD measurements

The CD signal of DARPin domains at 10 μM in PBS, pH 7.4 (PBS, 2 M GdmCl, pH 7.4, were indicated) were recorded at 222 nm in a Jasco J-810 instrument (Jasco) using a 1 mm pathlength cuvette. The samples were heated from 20 °C to 95 °C using a temperature ramp rate of 1 °C per min, collecting data periodically at 0.5 °C intervals. The melting temperature values were derived, as described by Consalvi *et al*. (37). Importantly, all constructs assessed in CD ran as monomeric peaks in analytical size-exclusion chromatography (aSEC).

### MD simulations

#### System preparation

Starting from the X-ray diffraction structure of ankyrin repeat proteins of E3_5, NI1C-mut4, and NI_3_C-mut5 (PDB ID: 1MJ0 (27), 2XEN (25), and 2XEE (25), respectively), homology modeling was used to construct atomic resolution models of six different repeat proteins, that is, N1C_v16, N1C_v17, N1C_v01, N1C_v05, N1C_v22, and N1C_v23. The sequences of the proteins are shown in Supplementary Table S1. These models were used as the structural basis of this study.

#### Simulation protocol

All simulations were carried out using the GROMACS 2018.6 simulation package (38) and the CHARMM36m force field (39). Three independent simulations with different initial random velocities were carried out for each repeat protein, two at 350 K and one at 400 K, cumulating 1.8 μs for each protein. To reproduce neutral pH conditions, standard protonation states were used for the ionizable side chains, the N-terminus was positively charged, and the C-terminus was negatively charged. Each protein was solvated in a cubic box (edge length of 6.9 nm) with TIP3P water molecules (40) to which 150 mM NaCl were added, including neutralizing counterions. After the steepest descent minimization, the simulation systems were first equilibrated under constant pressure for 5 ns, with position restraints applied on the heavy atoms and subsequently under constant temperature (T = 300 K) in the absence of restraints for 5 ns. For the production simulations, temperature and pressure were maintained constant at 350 K or 400 K and 1 atm, by using the modified Berendsen thermostat (0.1 ps coupling) (41) and barostat (2 ps coupling) (42). The short-range interactions were cutoff beyond a distance of 1.2 nm and the potential smoothly decays to zero using the Verlet cutoff scheme. The Particle Mesh Ewald technique (43) with a cubic interpolation order, a real space cut-off of 1.2 nm and a grid spacing of 0.16 nm, was used to compute the long-range interactions. The bond lengths were constrained using a fourth order LINCS algorithm (44) with two iterations. In all simulations, the time step was fixed to 4 fs, enabled through the use of virtual sites for all hydrogen atoms. Periodic boundary conditions were applied, and the snapshots were saved every 50 ps. The elevated temperature is used to enhance the sampling; the density of water is kept at the value corresponding to 300 K to perturb the free energy surface as little as possible (45).

## Data availability

The datasets generated and/or analyzed during the current study are available from the corresponding author upon reasonable request.

## Supporting information

This article contains supporting information.

## Conflict of interest

The authors declare that they have no conflicts of interest with the contents of this article.
