# Supplementary material for: Thermostable designed ankyrin repeat proteins (DARPins) as building blocks for innovative drugs
Source: J Biol Chem. 2021 Nov 15;298(1):101403. doi: 10.1016/j.jbc.2021.101403 (PMC8683736; doi:10.1016/j.jbc.2021.101403)
Supplement: Table S1 [file mmc1.pdf]

Supplementary Tables

Table S1. Protein sequences used in this study.

Name	Sequence
N1C_v01	GSDLGKKLLE AARAGQ**D**DEV RELLKAGADV NAKDKDGYTP LHLAAREGHL EIVEVLLKAG ADVNAQDKFG KTAFDISIDN GNEDLAEILQ KLN
N1C_v02	GSDAGKKLLE AARAGQ**D**DEV RELLKAGADV NAKDKDGYTP LHLAAREGHL EIVEVLLKAG ADVNAQDKFG KTAFDISIDN GNEDLAEILQ KLN
N1C_v03	GSDLAKKLLE AARAGQ**D**DEV RELLKAGADV NAKDKDGYTP LHLAAREGHL EIVEVLLKAG ADVNAQDKFG KTAFDISIDN GNEDLAEILQ KLN
N1C_v04	GSDLGKKLLE AARAGQ**A**DEV RELLKAGADV NAKDKDGYTP LHLAAREGHL EIVEVLLKAG ADVNAQDKFG KTAFDISIDN GNEDLAEILQ KLN
N1C_v05	GSDLGKKLLE AARAGQ**L**DEV RELLKAGADV NAKDKDGYTP LHLAAREGHL EIVEVLLKAG ADVNAQDKFG KTAFDISIDN GNEDLAEILQ KLN
N1C_v06	GSDLGKKLLE AARAGQ**V**DEV RELLKAGADV NAKDKDGYTP LHLAAREGHL EIVEVLLKAG ADVNAQDKFG KTAFDISIDN GNEDLAEILQ KLN
N1C_v07	GSDLGKKLLE AARAGQ**M**DEV RELLKAGADV NAKDKDGYTP LHLAAREGHL EIVEVLLKAG ADVNAQDKFG KTAFDISIDN GNEDLAEILQ KLN
N1C_v08	GSDLGKKLLE AARAGQ**I**DEV RELLKAGADV NAKDKDGYTP LHLAAREGHL EIVEVLLKAG ADVNAQDKFG KTAFDISIDN GNEDLAEILQ KLN
N1C_v09	GSDLGKKLLE AARAGQ**T**DEV RELLKAGADV NAKDKDGYTP LHLAAREGHL EIVEVLLKAG ADVNAQDKFG KTAFDISIDN GNEDLAEILQ KLN
N1C_v10	GSDLGKKLLE AARAGQ**S**DEV RELLKAGADV NAKDKDGYTP LHLAAREGHL EIVEVLLKAG ADVNAQDKFG KTAFDISIDN GNEDLAEILQ KLN
N1C_v11	GSDLGKKLLE AARAGQ**N**DEV RELLKAGADV NAKDKDGYTP LHLAAREGHL EIVEVLLKAG ADVNAQDKFG KTAFDISIDN GNEDLAEILQ KLN
N1C_v12	GSDLGKKLLE AARAGQ**Q**DEV RELLKAGADV NAKDKDGYTP LHLAAREGHL EIVEVLLKAG ADVNAQDKFG KTAFDISIDN GNEDLAEILQ KLN
N1C_v13	GSDLGKKLLE AARAGQ**K**DEV RELLKAGADV NAKDKDGYTP LHLAAREGHL EIVEVLLKAG ADVNAQDKFG KTAFDISIDN GNEDLAEILQ KLN
N1C_v14	GSDLGKKLLE AARAGQ**R**DEV RELLKAGADV NAKDKDGYTP LHLAAREGHL EIVEVLLKAG ADVNAQDKFG KTAFDISIDN GNEDLAEILQ KLN
N1C_v15	GSDLGKKLLE AARAGQ**E**DEV RELLKAGADV NAKDKDGYTP LHLAAREGHL EIVEVLLKAG ADVNAQDKFG KTAFDISIDN GNEDLAEILQ KLN
N1C_v16	GSDLGKKLLE AARAGQ**D**DEV RILMANGADV NAKDKDGYTP LHLAAREGHL EIVEVLLKAG ADVNAQDKFG KTAFDISIDN GNEDLAEILQ KLN
N1C_v17	GSDLGKKLLE AARAGQ**L**DEV RILMANGADV NAKDKDGYTP LHLAAREGHL EIVEVLLKAG ADVNAQDKFG KTAFDISIDN GNEDLAEILQ KLN
N1C_v19	DADLAKKLLA AAAAGQ**D**EAV EQLLKAGADV NAKDKDGYTP LHLAAREGHL EIVEVLLKAG ADVNAQDKFG KTAFDISIDN GNEDLAEILQ KLN
N1C_v20	DADLAKKLLA AAAAGQ**L**EAV EQLLKAGADV NAKDKDGYTP LHLAAREGHL EIVEVLLKAG ADVNAQDKFG KTAFDISIDN GNEDLAEILQ KLN
N1C_v22	GSDLGKKLLE AARAGQ**D**DEV RELLKAGADV NAKDKDGYTP LHLAAREGHL EIVEVLLKAG ADVNAQDKFG KTPFDLAIDN GNEDIAEVLQ KAA
N1C_v23	GSDLGKKLLE AARAGQ**L**DEV RELLKAGADV NAKDKDGYTP LHLAAREGHL EIVEVLLKAG ADVNAQDKFG KTPFDLAIDN GNEDIAEVLQ KAA
N1C_v25	GSDLGKKLLE AARAGQ**D**DEV RILMANGADV NAKDKDGYTP LHLAAREGHL EIVEVLLKAG ADVNAQDKFG KTPFDLAIDN GNEDIAEVLQ KAA
aHER2_v01	GSDLGKKLLE AARAGQ**D**DEV RILMANGADV NAKDEYGLTP LYLATAHGHL EIVEVLLKNG ADVNAVDAIG FTPLHLAAFI GHLEIAEVLL KHGADVNAQD KFGKTAFDIS IGNGNEDLAE ILQKLN
aHER2_v02	GSDLGKKLLE AARAGQ**L**DEV RILMANGADV NAKDEYGLTP LYLATAHGHL EIVEVLLKNG ADVNAVDAIG FTPLHLAAFI GHLEIAEVLL KHGADVNAQD KFGKTAFDIS IGNGNEDLAE ILQKLN

aVEGF_v01	GSDLDKKLLE	AARAGQ**D**DEV	RILMANGADV	NARDSTGWTP	LHLAAPWGHP	EIVEVLLKNG
	ADVNAADFQG	WTPLHLAAAV	GHLEIVEVLL	KYGADVNAQD	KFGKTAFDIS	IDNGNEDLAE
	ILQKAA					
aVEGF_v02	GSDLDKKLLE	AARAGQ**L**DEV	RILMANGADV	NARDSTGWTP	LHLAAPWGHP	EIVEVLLKNG
	ADVNAADFQG	WTPLHLAAAV	GHLEIVEVLL	KYGADVNAQD	KFGKTAFDIS	IDNGNEDLAE
	ILQKAA					
aHSA_v01	GSDLGKKLLE	AARAGQ**D**DEV	RELLKAGADV	NAKDYFSHTP	LHLAARNGHL	KIVEVLLKAG
	ADVNAKDFAG	KTPLHLAANE	GHLEIVEVLL	KAGADVNAQD	IFGKTPADIA	ADAGHEDIAE
	VLQKAA					
aHSA_v02	GSDLGKKLLE	AARAGQ**L**DEV	RELLKAGADV	NAKDYFSHTP	LHLAARNGHL	KIVEVLLKAG
	ADVNAKDFAG	KTPLHLAANE	GHLEIVEVLL	KAGADVNAQD	IFGKTPADIA	ADAGHEDIAE
	VLQKAA					
